# Polymorphic genetic characterization of the ORF7 gene of porcine reproductive and respiratory syndrome virus (PRRSV) in China

**DOI:** 10.1186/1743-422X-8-73

**Published:** 2011-02-19

**Authors:** Xiaofang Hao, Zengjun Lu, Wendong Kuang, Pu Sun, Yu Fu, Lei Wu, Qing Zhao, Huifang Bao, Yuanfang Fu, Yimei Cao, Pinghua Li, Xingwen Bai, Dong Li, Zaixin Liu

**Affiliations:** 1 State Key Laboratory of Veterinary Etiological Biology, Key Laboratory of Animal Virology of the Ministry of Agriculture, Lanzhou Veterinary Research Institute, Chinese Academy of Agricultural Sciences, No. 1 Xujiaping, Yanchangbao, Lanzhou, Gansu 730046, PR China

## Abstract

**Background:**

Porcine reproductive and respiratory syndrome virus (PRRSV) exhibits extensive genetic variation. The outbreak of a highly pathogenic PRRS in 2006 led us to investigate the extent of PRRSV genetic diversity in China. To this end, we analyzed the Nsp2 and ORF7 gene sequences of 98 Chinese PRRSV isolates.

**Results:**

Preliminary analysis indicated that highly pathogenic PRRSV strains with a 30-amino acid deletion in the Nsp2 protein are the dominant viruses circulating in China. Further analysis based on ORF7 sequences revealed that all Chinese isolates were divided into 5 subgroups, and that the highly pathogenic PRRSVs were distantly related to the MLV or CH-1R vaccine, raising doubts about the efficacy of these vaccines. The ORF7 sequence data also showed no apparent associations between geographic or temporal origin and heterogeneity of PRRSV in China.

**Conclusion:**

These findings enhance our knowledge of the genetic characteristics of Chinese PRRSV isolates, and may facilitate the development of effective strategies for monitoring and controlling PRRSV in China.

## Background

Porcine reproductive and respiratory syndrome (PRRS) is a severe disease characterized by reproductive disorders in gilts and sows, especially during late gestation, and by respiratory distress in pigs. The disease first emerged in late 1987 in the United States [1] and three years later in Europe [2]. Porcine reproductive and respiratory syndrome virus (PRRSV), the causative agent of PRRS, belongs to the family *Arteriviridae *in the order *Nidovirales *[3], and is an enveloped virus with a single-stranded positive sense RNA genome containing nine open reading frames (ORFs) [4]. The ORFs 1a and 1b encode the non-structural proteins Nsp1a, Nsp1b, and Nsp2-12, while ORF2a, ORF2b, and ORFs 3-7 encode the structural proteins GP2a, GP2b, GP3, GP4, GP5, M, and N, respectively [5].

Two genotypes are recognized for PRRSV, the North American type and the European type, as represented by the prototypes VR-2332 and Lelystad virus (LV), respectively [6]. Significant genetic differences have been described both between these two genotypes and within the same genotype of PRRSV [7-9].

Since the first report of PRRSV in China in 1996 [10], the North American type PRRSV, with considerable genetic variation, has spread throughout the country [11-13]. Since 2006, several highly pathogenic PRRS outbreaks have been reported in China, causing severe economic losses in the swine industry [14-16]. The molecular characterization of these PRRSVs is thus a major focus of Chinese virology research [17-19]. However, previous studies have focused mainly on the ORF5 or Nsp2 genes, while the genetics of the Chinese PRRSVs based on the ORF7 gene are not well characterized. The ORF7 encodes the nucleocapsid protein (N), the most abundant viral protein in virus-infected cells [5] and the most immunodominant antigen in the pig immune response to PRRSV [20]. ORF7 is, therefore, a promising candidate for serological detection and diagnosis [21,22]. Indeed, the N protein has been extensively used for determining the genetic variation and the phylogenetic relationships among PRRSVs [23-25], suggesting a significant role for ORF7 in PRRSV evolutional surveillance.

In this paper, we sequenced the ORF7 and Nsp2 genes of seven PRRSV strains isolated during the outbreak of highly pathogenic PRRS in China. We analyzed the ORF7-coding region to assess the genetic variation of PRRSV in China and to better understand the molecular epidemiology of PRRS.

## Results

### The genetic diversity of the Nsp2 gene among Chinese PRRSV isolates

To determine whether the isolates examined in this study possessed the same characteristic deletions in the Nsp2 gene found in the highly pathogenic PRRSVs previously described [14,15], the partial Nsp2 sequences of all 7 PRRSV isolates were sequenced and aligned with the 93 isolates in GenBank. The GenBank sequences included 91 Chinese reference isolates, in addition to the North American prototype VR-2332 and its attenuated vaccine virus RespPRRS MLV (Table 1). Alignment analysis of the deduced amino acid sequences revealed that 23 Chinese isolates, including our XJ isolate, contained no deletions or insertions in comparison to VR2332 and RespPRRS MLV. In contrast, deletions occurred within the Nsp2 protein in 75 Chinese strains. Of these, 74 were isolated during the outbreak of highly pathogenic PRRSV (except for the stain HB-2sh collected in 2002). The HB-2sh strain contains a continuous 12 amino acid deletion at position 470-481, while the other 74 isolates have four different types of deletions. Sixty-four Chinese reference isolates and 6 of our isolates (07N, 128, PC, TS, XIN, and XB) contained a discontinuous deletion of 1 and 29 amino acids at positions 482 and 533-561. In addition, CG and GDQY2 contained a discontinuous deletion of 36 and 29 amino acids at positions 471-506 and 533-561. The YN9 strain contained a discontinuous deletion of 25 and 29 amino acids at positions 478-502 and 533-561. The Em2007 strain had a continuous deletion of 68 amino acids at position 499-566. Therefore, the highly pathogenic PRRS viruses with a discontinuous 30 amino acid deletion have been the dominant strains circulating in China.

**Table 1:** Details of the PRRSV isolates examined in this report.

**No**.	Isolate	Province	Date of isolation	**GenBank accession No**.	**No**.	Isolate	Province	Date of isolation	GenBank accession **No**.
1	CH-1a	Beijing	1996	AY032626	51	CG	Guangdong	2007	EU864231
2	BJ-4	Beijing	1996	AF331831	52	Em2007	Hubei	2007	EU262603
3	S1	Henan	1998	DQ459471	53	GD	Guangdong	2007	EU109503
4	PRRSV01	Gansu	2001	FJ175687	54	GD	Guangdong	2007	EU825724
5	HB-1sh	Hebei	2002	AY150312	55	GD2007	Guangdong	2007	EU880433
6	HB-2sh	Hebei	2002	AY262352	56	GDQJ	Guangdong	2007	GQ374441
7	CH2002	Gansu	2002	EU880438	57	GDQY2	Guangdong	2007	GU454850
8	GS2002	Gansu	2002	EU880441	58	Henan-1	Henan	2007	EU200962
9	PRRSV02	Gansu	2002	FJ175688	59	HN2007	Henan	2007	EU880437
10	CH2003	Gansu	2003	EU880440	60	HPBEDV	Gansu	2007	EU236259
11	Clone20	Hubei	2003	FJ899592	61	Jiangxi-3	Jiangxi	2007	EU200961
12	GS2003	Gansu	2003	EU880442	62	LN	Liaoning	2007	EU109502
13	HN1	Henan	2003	AY457635	63	NM1	Jilin	2007	EU860249
14	PRRSV03	Gansu	2003	FJ175689	64	SHH	Shanghai	2007	EU106888
15	CH2004	Gansu	2004	EU880439	65	SX2007	Shanxi	2007	EU880434
16	GS2004	Gansu	2004	EU880443	66	XH-GD	Guangdong	2007	EU624117
17	NB04	Zhejiang	2004	FJ536165	67	08HuN	Hunan	2008	GU169411
18	GD3	Guangdong	2005	GU269541	68	08SDWF	Shandong	2008	GU168569
19	SHB	Guangdong	2005	EU864232	69	CBB-1-F3	Chongqing	2008	FJ889129
20	BJsy06	Beijing	2006	EU097707	70	CH-1R	Beijing	2008	EU807840
21	CC-1	Jilin	2006	EF153486	71	CWZ-1-F3	Chongqing	2008	FJ889130
22	HEB1	Hebei	2006	EF112447	72	GDBY1	Guangdong	2008	GQ374442
23	HN-HW	Hunan	2006	FJ797690	73	GS2008	Gansu	2008	EU880431
24	HUB1	Hubei	2006	EF075945	74	JN-HS	Shandong	2008	HM016158
25	HUB2	Hubei	2006	EF112446	75	KP	Guangdong	2008	GU232735
26	HuN	Hunan	2006	EF517962	76	SD-CXA2008	Shandong	2008	GQ359108
27	HUN4	Hunan	2006	EF635006	77	WUH2	Hubei	2008	EU678352
28	JSyx	Jiangsu	2006	EU939312	78	WUH3	Hubei	2008	HM853673
29	JX0612	Jiangxi	2006	EF488048	79	XL2008	Gansu	2008	EU880436
30	JX143	Jiangxi	2006	EU708726	80	YN2008	Yunnan	2008	EU880435
31	JX2006	Jiangxi	2006	EU880432	81	YN9	Yunnan	2008	GU232738
32	JXA1	Jiangxi	2006	EF112445	82	09HUB5	Hubei	2009	GU168568
33	JXwn06	Jiangxi	2006	EF641008	83	09HUB7	Hubei	2009	GU168567
34	NX06	Ningxia	2006	EU097706	84	GX09-16	Guangxi	2009	HM214913
35	SY0608	Jiangsu	2006	EU144079	85	GX09-29	Guangxi	2009	HM214914
36	TJ	Jilin	2006	EU860248	86	GX09-32	Guangxi	2009	HM214915
37	TP	Guangdong	2006	EU864233	87	HLJHL	Heilongjiang	2009	HM189676
38	WUH1	Hubei	2006	EU187484	88	SD1-100	Shandong	2009	GQ914997
39	07BJ	Beijing	2007	FJ393459	89	SX-1	Shandong	2009	GQ857656
40	07HEBTJ	Hebei	2007	FJ393458	90	SX2009	Shanxi	2009	FJ895329
41	07HEN	Henan	2007	FJ393457	91	ZP-1	Shandong	2009	HM016159
42	07NM	Neimenggu	2007	FJ393456	92	07N	Gansu	2007	
43	AH0701	Anhui	2007	GU461292	93	128	Gansu	2008	
44	BJ	Beijing	2007	EU825723	94	XIN	Gansu	2009	
45	BJ0706	Beijing	2007	GQ351601	95	PC	Gansu	2009	
46	BJBLZ	Beijing	2007	FJ950745	96	TS	Gansu	2010	
47	BJPG	Beijing	2007	FJ950746	97	XB	Gansu	2010	
48	BJSD	Beijing	2007	FJ950747	98	XJ	Gansu	2010	
49	BJSY07	Beijing	2007	HM011104	99	VR-2332			PRU87392
50	BJSY-1	Beijing	2007	FJ950744	100	RespPRRSMLV			AF066183

Note:

1-91: Reference Chinese isolates in GenBank

92-98: Isolates sequenced in this study

99-100: The North American prototype virus VR-2332 and its attenuated vaccine stain RespPRRS MLV

### Phylogenetic analysis based on the ORF7 sequence

In order to better understand the genetic relationship and evolution of PRRSVs in China from 1996 to 2010, phylogenetic analysis was carried out based on the ORF7 gene sequences of our 7 isolates and the 93 reference viruses in GenBank (VR-2332, RespPRRS MLV, and 91 Chinese reference isolates).

**Figure 1:**
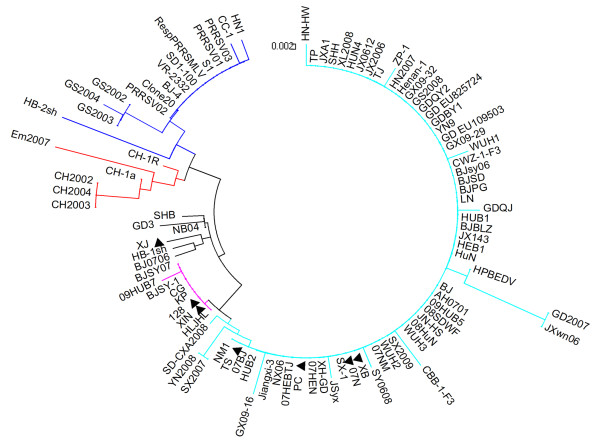
**Phylogenetic relationship between 98 Chinese isolates, the prototype North American strain VR-2332, and its attenuated RespPRRS MLV vaccine stain based on the amino acid sequences of the ORF7 gene**. A neighbor-joining tree was constructed with bootstrap values calculated from 1,000 replicates. The five subgroups are marked with different colors and the strains isolated in this study are indicated by a filled triangle.

In the phylogenetic tree, all these PRRSV isolates were divided into 5 subgroups (Figure 1). Subgroup I was comprised of 15 isolates that included VR-2332, RespPRRS MLV, and 13 Chinese reference isolates. The isolates within subgroup I showed 92.7-100% identity at the nucleotide level and 92.7-100% at the amino acid level. Subgroup II consisted of 6 PRRSV reference isolates, Em2007, CH-1a, and the 4 CH-1a derivatives (CH2002, CH2003, CH2004, and CH-1R). In this subgroup, sequence identities were 94.9-100% at the nucleotide level and 94.3-100% at the amino acid level. Subgroup III consisted of 6 PRRSV isolates, including our XJ isolate. The other 5 isolates were BJ0706, HB-1sh, GD3, NB04, and SHB. Within subgroup III, nucleotide and amino acid sequence identities were 95.7-98.4% and 96.7-99.2%, respectively.

Subgroup IV contained 2 of our isolates (128 and XIN) and 6 highly pathogenic reference viruses (09HUB7, BJSY07, BJSY-1, CG, HLJHL, and KP). They shared 98.4-100% nucleotide sequence identity and 99.2-100% amino acid sequence identity. Subgroup V consisted of 65 isolates, including 4 of our isolates (07N, PC, TS, and XB) and 61 highly pathogenic reference isolates. Their nucleotide and amino acid sequences were 95.7-100% and 94.3-100% identical. Highly pathogenic PRRSVs were concentrated in Subgroups IV and V, except for the strain Em2007, which was located in subgroup II and considered a recombinant between the vaccine strain CH-1R and the highly pathogenic virus [26].

The identities among the 98 Chinese isolates (our 7 isolates and 91 Chinese reference isolates) in all the 5 subgroups ranged from 90.9% to 100% for the nucleotide sequences and from 88.6% to 100% for the amino acid sequences. All 98 Chinese isolates were of the North American genotype. These Chinese isolates demonstrated 91.4-100% nucleotide sequence identity and 91.1-100% amino acid sequence identity with the North American prototype VR-2332 and its derived vaccine virus RespPRRS MLV (VR-2332 and RespPRRS MLV have identical ORF7 sequences). In contrast, the Chinese isolates had 67.8-71.1% nucleotide sequence identity and 62.5-65.8% amino acid sequence identity with the Lelystad virus (the European prototype).

### Sequence comparison of ORF7 gene among Chinese isolates

**Figure 2:**
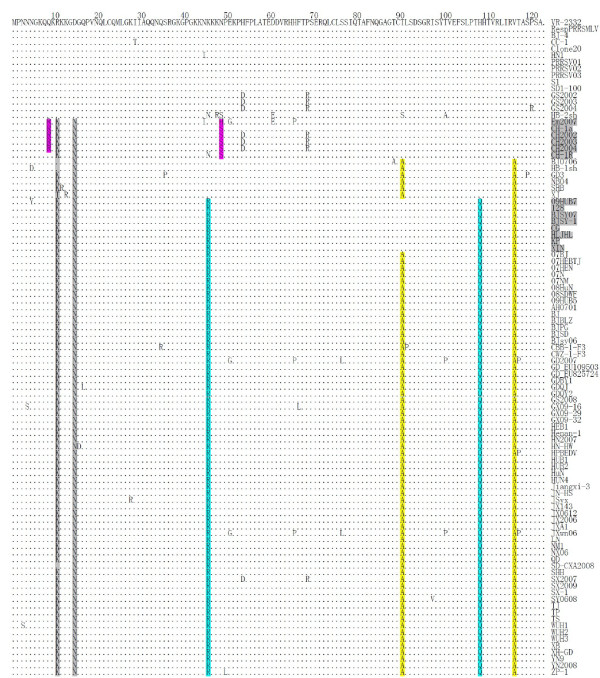
**Alignment of the deduced amino acid sequences of the ORF7 gene of 98 Chinese isolates and 2 American reference strains (VR-2332 and MLV)**. Dots (.) indicate the same amino acids as in VR2332, and substitutions are indicated by the amino acid letter codes.

The ORF7 sequences of the 5 subgroups, including 98 Chinese isolates and the 2 foreign isolates (VR-2332 and RespPRRS MLV), were further compared and analyzed. As shown in Figure 2 all of the PRRSV ORF7 sequences were the same length (372 nt), and encoded 123 amino acid residues. Many amino acid substitutions were observed in subgroups II, III, IV, and V when compared with VR-2332. Some substitutions, such as R11K and D15N, occurred in subgroups II, III, IV, and V. The V117A substitution was unique to subgroups III, IV, and V, while T91A was unique to subgroups III and V. Interestingly, two substitutions, K46R and H109Q, were exclusive to all isolates in subgroups IV and V, the most highly pathogenic PRRSVs. In addition, subgroup II strains had two distinct substitutions from the other subgroups, Q9R and N49 S, with the exceptions of CH-1a (no substitution occurred at amino acid residue 9) and Em2007 (N49H).

**Figure 3:**
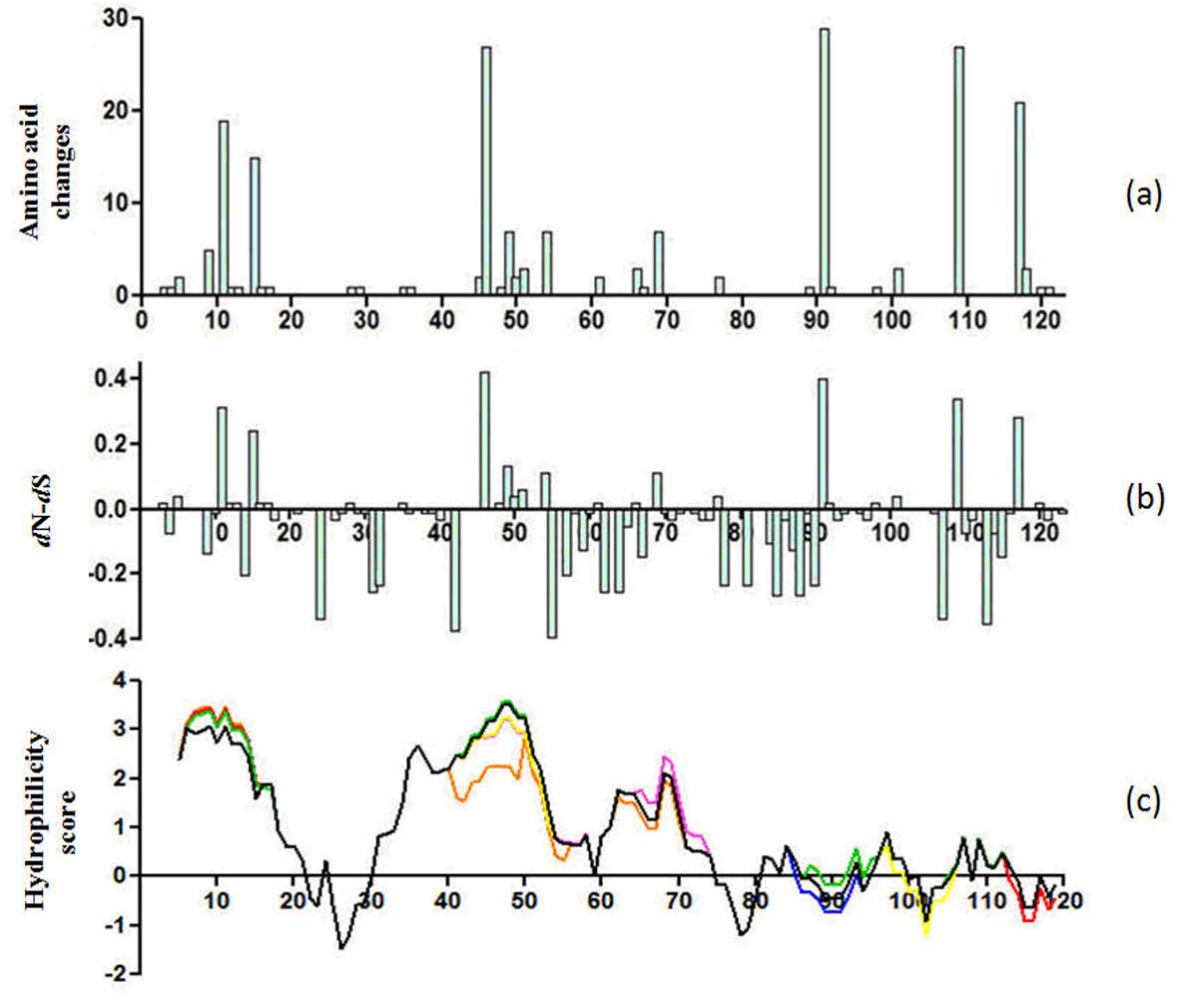
**(a) The distribution of the amino acid mutations along the N protein of the 100 isolates analyzed in the study**. (b) Differences between non-synonymous and synonymous substitutions (dN-dS) for ORF7 sequences of all the 100 isolates. (c) Hydrophilicity profiles of the N proteins of representative PRRSVs.

The distribution of sequence diversity across the ORF7 protein was investigated for all 100 sequences analyzed in this study. These sequences contained 108/372 (29.0%) polymorphic nucleic acid positions and 36/123 (29.6%) polymorphic amino acid positions. However, frequent amino acid alterations were only found in the individual residues 11, 15, 46, 91, 109, and 117, while conserved regions were present primarily at positions 16-45, 55-90, and 92-108 (Figure 3a). Figure 3b shows the amino acid positions plotted versus the difference between non-synonymous and synonymous substitution rates (dN-dS). The overall difference between dN and dS for ORF7 was negative (-0.03258 ± 0.01219), indicating that ORF7 was under purifying selection. Most negative values were located mainly in the middle domain (residues 55-90) of the protein. However, some residues were under positive selection, such as the above-mentioned six amino acid sites 11, 15, 46, 91, 109, and 117.

Hydrophilic analysis was performed using at least three isolates in each subgroup. Figure 3c shows the ORF7 hydrophilicity plots of isolates VR2332, BJ-4, and HB-2sh in subgroup I, CH-1a, CH2003, and Em2007 in subgroup II, BJ0706, HB-1sh, and XJ in subgroup III, CG, 128, and XIN in subgroup IV, and HUN4, JXA1, and XB in subgroup V. The hydropathy profiles of the PRRSV ORF7 s indicated that the proteins were highly hydrophilic, especially in the N-terminal domain, with two large hydrophilic regions at positions 5-21 and 30-74. The C-termini also possessed several small hydrophilic regions at 81-85, 95-99, and 106-112. The differences in hydrophilicity plots between these isolates were centralized in the segments 7-15, 41-55, 62-73, 85-105, and 113-120, and all resulted from single amino acid substitutions within these regions.

## Discussion

Porcine reproductive and respiratory syndrome virus (PRRSV) has been one of the most economically damaging pathogens for the swine industry world-wide. Since it first emerged in 1996, the virus has spread widely throughout pig-producing provinces of China, imposing a considerable economic burden on the swine industry, especially after the outbreak of highly pathogenic PRRS in 2006 [14,15]. Studies have been performed on genetic variability of these isolates, revealing extensive sequence variation among the Chinese PRRSV strains [17,18]. Nevertheless, the molecular characterization of the ORF7 gene among PRRSVs circulating in China has not been documented. Here, we determined the partial Nsp2 sequences and the complete ORF7 sequences of 7 PRRSV strains isolated in different swine herds during 2007-2010 and compared them with the published sequences of 91 Chinese strains and 2 North American strains (VR-2332 and its attenuated vaccine virus RespPRRS MLV).

Pair wise comparisons showed that 6 isolates characterized in this study (07N, 128, PC, TS, XIN, and XB) and 64 Chinese reference isolates contained a discontinuous deletion of 30 amino acids in the Nsp2 gene. The deletion is considered a gene marker for highly pathogenic PRRSV; however, it is not related to virulence [27]. Further analysis of the complete ORF7 sequences revealed that all the 98 Chinese PRRSV isolates analyzed exhibited a very high degree of genetic diversity, and clustered into 5 subgroups, suggesting the coexistence of related non-identical PRRS viral variants evolving independently. Subgroup I isolates shared a high identity with the MLV vaccine and its parent virus VR-2332. Subgroup II isolates were highly homologous to the CH-1R vaccine and its parent virus CH-1a. Isolates in subgroups IV and V were all highly pathogenic PRRSVs, and distinct from the MLV or CH-1R vaccine. These highly pathogenic subgroups IV and V PRRSVs are the dominant strains circulating in China, and so should be the focus when formulating preventive and control measures against PRRSV.

No apparent relationship between geographic and genetic distance was found for the isolates based on the N protein in the study, especially for the highly pathogenic strains, since these atypical viruses existed throughout the mainland of China. A correlation between temporal and genetic distance was also not found, as the highly pathogenic PRRS outbreak occurred in several pig farms in the summer of 2006 and rapidly spread to almost all pig-producing provinces of China. Our data indicate that the disease is still circulating in China.

The nucleocapsid protein (N) encoded by ORF7 is highly immunogenic, and several antigenic domains have been mapped onto N in both the European and the North American PRRSV. A common linear epitope conserved among different isolates of European and North American origin was located in the amino acid segment 50-66 [28]. Another linear epitope, conserved in European and North American isolates, was identified in amino acids 25-30 [29]. Wootton et al. found three additional linear epitopes (residues 30-52, 37-52, and 69-123) and one discontinuous epitope utilizing residues 52-69 and 112-123 [30]. In addition, four other linear epitopes at 23-33, 30-48, 30-50, and 43-56 were observed in VR2332 [31]. For the North American isolate CH-1a, the first Chinese isolate, epitopes were reported in amino acid segments 51-58 and 79-87 [32,33].

Extensive substitutions were observed in the 123-residue nucleocapsid protein on the basis of the alignment. Substitutions K46R and V117A occurred in all the highly pathogenic PRRSVs, which might impede the recognition of the epitopes encompassing or flanking the two substitutions by anti-N mAbs. A previous study confirmed that the 11 C-terminal residues 112-123 were essential for the generation of discontinuous epitopes [30]. Single amino acid substitutions introduced into the C-terminal domain show that the requirement of the C terminus for conformation-dependent mAb binding correlates with the proper formation of the predicted beta-strand formed by amino acids 111-117 [34]. Therefore, it is likely that the mutation V117A observed in highly pathogenic PRRSVs could exert great influence on the structure and antigenicity of N protein.

The substitution K46R might also alter the function of the nuclear localization signal (NLS) motif (41-47) and the nucleolar localization signal (NoLS) motif (41-72) [35]. Previous studies have demonstrated that mutations at 43 and 44 within the NLS attenuated viral replication [36]. Whether the mutation at amino acid position 46 has the similar or opposite impact on viral pathogenicity remains to be determined.

Two other substitutions, R11K and D15N, occurred in subgroups II, III, IV, and V, although there are a large number of Lys (K) and Asn (N) residues in the N-terminal half of the 123-residue nucleocapsid protein. The accumulation of these residues in the N terminus might function in the interaction with genomic viral RNA [37].

Some conserved sites were also observed from our alignment analysis. For example, three cysteine residues at amino acid positions 23, 75, and 90 were highly conserved in all isolates. Covalent interactions were formed through disulfide linkages between conserved cysteines at position 23 in North American strains, while the domain 30-37, which was also conserved in all isolates in this study, was shown to be essential for non-covalent interactions [38]. The C75 S mutant induced cytopathic effects and produced infectious strains with plaque morphology indistinguishable from the wild type clone. In contrast, the C23 S and C90 S mutations completely abolished viral infectivity, indicating that C23 and C90 play critical roles in PRRSV infection [39].

Conserved regions were also found by variability analysis at positions 16-45, 55-90, and 92-108. These highly conserved amino acid segments are probably associated with nucleocapsid structure and/or function. Meanwhile, non-synonymous mutations did not occur more frequently than synonymous mutations among the Chinese isolates, and the main variable sites and non-synonymous mutations (residues 11, 15, 46, 91, 109, and 117) were distributed in the hydrophilic regions prone to immune pressure.

The N protein contains important immunogenic epitopes, and the majority of antibodies produced during PRRSV infection are specific for it [20,40]. Thus, the N protein has been targeted as a suitable candidate for the detection and diagnosis of PRRS. Numerous serological diagnostic tests have been developed based on the N protein [21,22,41-43]. Additionally, PCR is another widely used method for detecting the viruses, and ORF7 has been regarded as a promising target gene [25,44,45] due to its sequence stability relative to other structural genes [46]. However, the high genetic variability among the ORF7 sequences of the Chinese PRRSV isolates observed in this study should be taken into consideration when designing serological or molecular detection methods for PRRSV diagnosis and epidemiological surveillance.

In conclusion, all the ORF7 sequences of PRRSV isolates from 1996 to 2010 in China belonged to the North American type. Chinese strains were categorized into five subgroups. The highly pathogenic PRRSVs have become the dominant strains in China. Our study provides the first genetic analysis of the Chinese PRRSV N protein. These results could lead to a better understanding of the molecular variation of PRRSV in China and to the development of more effective vaccines and reliable diagnostic methods.

## Materials and methods

### Sample origin

Fresh tissues were sampled during 2007-2010 from different swine herds in Gansu province of northwestern China. This region experienced outbreaks of severe reproductive failure in pregnant sows and respiratory problems in sucking and post-weaning piglets. All samples were stored in ice boxes and transported to the laboratory immediately. The materials were frozen at -80°C until analyzed.

### RNA extraction and RT-PCR

Total RNA was extracted from the tissue homogenates using Qiagen RNeasy Mini kit (Qiagen, Hilden, Germany). Viral cDNA was synthesized using Oligo dT primer according to the manufacturer's instructions (TaKaRa, Dalian, China). The sense primer for Nsp2 amplification was 5'-AGGAAGGTCAGATCCGATTG-3' and the reverse primer was 5'-CGTCTGTGAGGACGCAGACA-3'. The cycling conditions were 95°C for 4 min, followed by 30 cycles of 94°C for 1 min, 58°C for 30 sec, and 72°C for 30 sec, and a final extension at 72°C for 10 min. This yielded a 370 bp fragment of the Nsp2 gene. For ORF7 gene amplification, the sense primer was 5'-AAGCCTCGTGTTGGGTGGCAG-3' and the antisense primer was 5'-TCTCCCAATTCTAACACTGAG-3'. The PCR protocol included 95°C for 4 min and then 30 cycles of 94°C for 1 min, 56°C for 30 sec, 72°C for 30 sec, followed by a final extension at 72°C for 10 min. Amplification yielded the complete sequence of the ORF7 gene.

### Nucleotide sequencing

The PCR products were purified using a PCR purification kit (Axygen, USA) and cloned into the pMD18-T vector (TaKaRa, China). At least three clones were generated from each cDNA fragment and used for sequencing.

### Data analysis

Sequence analysis of the partial Nsp2 gene and the complete ORF7 gene from all 7 isolates found in this study, in addition to 93 reference isolates from GenBank (Table 1), was conducted using the Lasergene sequence analysis software package (DNASTAR Inc., Madison, WI). The CLUSTAL W program was used for multiple sequence alignment. The unrooted phylogenetic tree was generated by the distance-based neighbor-joining method using MEGA 4.0. Boot-strap values were calculated using 1000 replicates of the alignment. A hydrophilicity profile was generated with the ProtScale web utility http://expasy.org/tools/protscale.html by the Kyte and Doolittle method. Furthermore, the amino acid position was plotted versus the difference between non-synonymous and synonymous substitution rates (*d*N-*d*S). The difference was calculated with the SNAP web utility http://hiv-web.lanl.gov/content/hiv-db/SNAP/WEBSNAP/SNAP.html.

## Competing interests

The authors declare that they have no competing interests.

## Authors' contributions

ZJL and ZXL were responsible for the research design. XFH, WDK, PS, YF, LW, QZ, HFB, YFF, YMC, PHL, XWB, and DL carried out the experiments and wrote the manuscript. All authors read and approved the final manuscript.
